# *TERT* rs2736098 (Ex2-659G>A) polymorphism and cancer susceptibility: evidence from a comprehensive meta-analysis

**DOI:** 10.18632/oncotarget.21703

**Published:** 2017-10-09

**Authors:** Tingyuan Pang, Minjie Zhou, Rumin Liu, Jia Luo, Renfei Xia

**Affiliations:** ^1^ Department of Pharmacy, Affiliated Cancer Hospital & Institute of Guangzhou Medical University, Guangzhou City, 510000, P.R. China; ^2^ Department of Kidney Transplantation, Nanfang Hospital, Southern Medical University, Guangzhou City, 510000, P.R. China

**Keywords:** TERT, rs2736098, polymorphism, cancer, meta-analysis

## Abstract

Increasing researches have been performed regarding the relationship between *TERT* rs2736098 and cancer risk, but no consensus has been reached about the relationship. Here, we conducted this updated meta-analysis, aiming to comprehensively evaluate the role of *TERT* rs2736098 in cancer risk. We systematically searched potential relevant articles through PubMed, EMBASE, CNKI, and WanFang database before August 2017. A total of 33 studies with 18685 cases and 23820 controls were finally included in the current meta-analysis. We then adopted odds ratios (ORs) and 95% confidence intervals (CIs) to analyze the contributions of *TERT* rs2736098 to cancer risk. We found that the *TERT* rs2736098 polymorphism was associated with risk of cancer in overall analysis (AA vs. GG: OR = 1.26, 95% CI = 1.09–1.47; AA vs. AG/GG: OR = 1.22, 95% CI = 1.09–1.36; AA/AG vs. GG: OR = 1.13, 95% CI = 1.02–1.24; A vs. G: OR = 1.11, 95% CI = 1.04–1.20). Furthermore, in analysis stratified by cancer type, ethnicity, control source, quality score, and Hardy-Weinberg equilibrium (HWE) in controls, we found increased risk of cancer among lung cancer, bladder cancer, breast cancer, colorectal cancer, other cancers, Asians, hospital-based subgroup, score > 9 group, as well as controls agreement with HWE group. Despite some limitations, the current meta-analysis represented the largest and the most comprehensive investigations, with the strongest conclusion than ever before. To further explicit the association between *TERT* rs2736098 and cancer risk, more well-design case-control studies with larger sample size are warranted in the future.

## INTRODUCTION

Cancer is a substantial public health burden, with an estimate of 14.1 million new cancer cases and 8.2 million cancer-related deaths occurred globally [1]. Although progress has been achieved in understanding the etiology of carcinogenesis, the definitive etiology still remains not yet fully elucidated. Mounting evidences have suggested that cancer is a multifactorial disease caused by genetic and environmental interactions [2–4].

Telomerase is an RNA-dependent DNA polymerase containing two essential components, catalytic subunit with the reverse transcriptase activity and an essential structural RNA component with a sequence complementary to the telomere sequence [5]. *TERT* gene is located on the short (p) arm of chromosome 5 at position 15.33 (5p15.33), and composes of 16 exons [6, 7]. *TERT* gene encodes the reverse transcriptase component of the telomerase, which is essential in maintaining the length of telomer [8]. In addition, telomerase is also responsible for chromosomal stability, and cellular immortality [9]. Telomeres might become shorter during mitosis due to incomplete replication of linear chromosomes by conventional DNA polymerases [10]. Normally, *TERT* mRNA is not expressed in most human somatic cells; however, aberrant expression of *TERT* mRNA and protein are associated with development of various cancers [11, 12].

More and more epidemiological studies were accessible regarding the association between the *TERT* rs2736098 polymorphism and cancer risk, yet conflicting conclusions remain. Besides, the latest meta-analysis was performed a year ago, which updated to March 2015. Nearly 10 new case-control studies with larger sample size were published since then. Thus, it is of great value to updated the meta-analysis regarding the association of interest. The current meta-analysis was the most comprehensive to date, which undoubtedly will shed some light on the current uncertain claims.

## RESULTS

### Study characteristics

**Figure 1:**
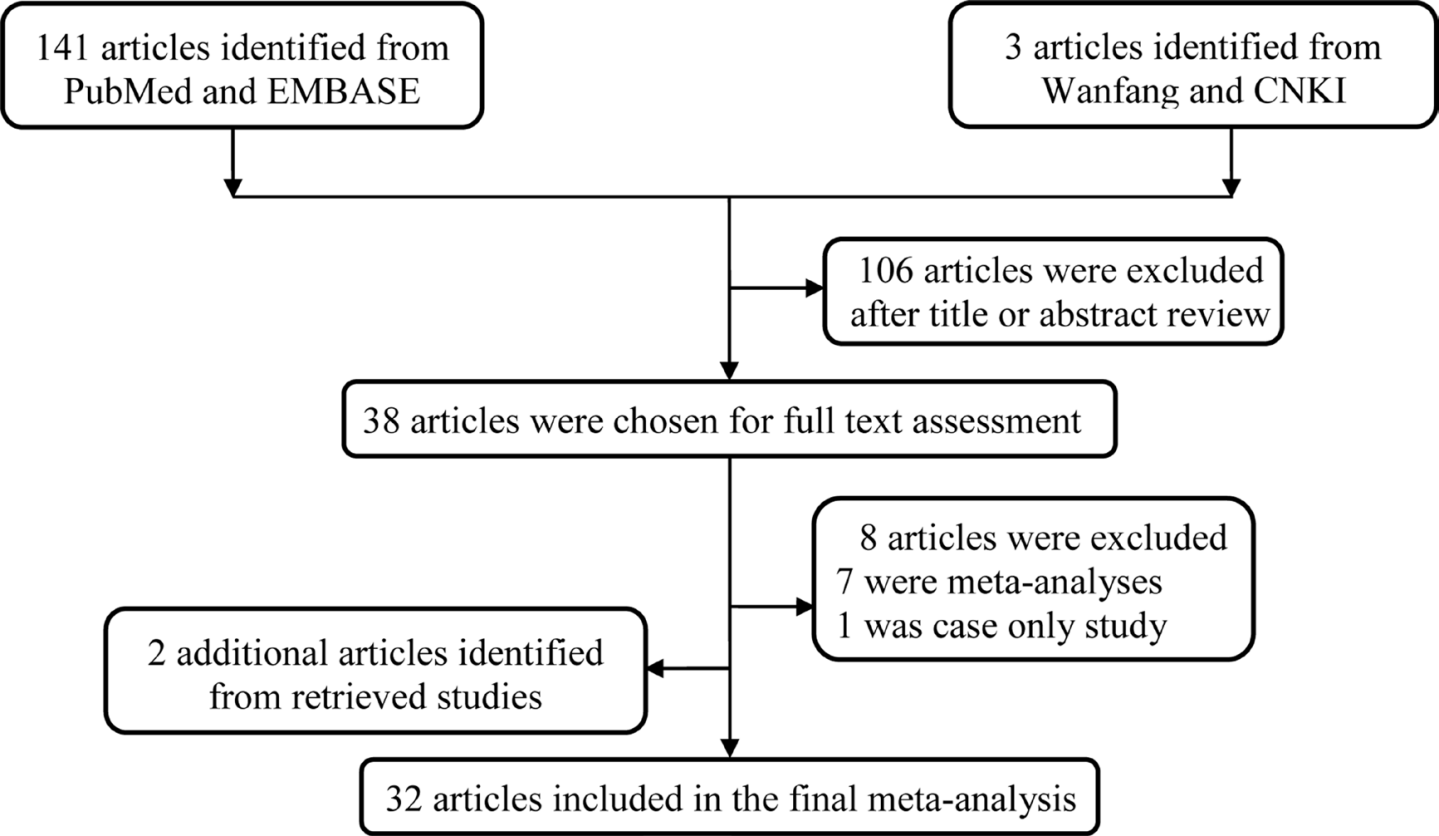
Flowchart of included studies

A total of 144 potentially relevant publications were initially identified from the databases. After screening titles and abstracts, 106 publications were excluded because of their failure to reach inclusion criteria. The remaining 38 publications were further assessed through careful reading. We further excluded 8 publications based on the following reasons: 7 publications were meta-analyses [13–19], 1 was case only research [20]. 2 additional publications were further extracted by manually screening the references of the retrieval articles [21, 22]. As a result, 33 studies including 32 publications were used for investigation [13, 16, 21–50]. The general workflow of selecting the eligible studies was graphically shown in Figure 1.

In general, the current study contains 18685 cases and 23820 controls (Table 1). Studies were conducted on several cancer types, including lung cancer, bladder cancer, breast cancer, colorectal cancer, cervical cancer, glioma, hepatocellular carcinoma (HCC), oral squamous cell carcinoma (OSCC), acute lymphoblastic leukemia (ALL), renal cell carcinoma (RCC), squamous cell carcinoma of head and neck (SCCHN), pancreatic cancer, esophageal carcinoma. In terms of ethnicities, 23 studies focused on Asians and 10 on Caucasians. Of these, there were 24 hospital based and 9 population based data sets. 18 studies were categorized as low quality and 15 were high quality. The controls’ genotype frequencies in agreement with Hardy-Weinberg equilibrium (HWE) was observed in 27 studies, while not available in 6 studies.

**Table 1:** Characteristics of studies included in the current meta-analysis

Surname	Year	Cancer type	Country	Ethnicity	Control Source	Genotype method	Genotype quality	Case				Control				HWE	Score
								GG	AG	AA	All	GG	AG	AA	All		
Savage	2007	Breast	Poland	Caucasian	PB	TaqMan	High	1171	699	97	1967	1313	811	141	2265	0.294	13
Choi	2009	Lung	Korea	Asian	HB	PCR-RFLP	High	311	322	87	720	345	320	55	720	0.101	11
Liu	2010	SCCHN	USA	Caucasian	HB	TaqMan	High	588	419	72	1079	576	461	78	1115	0.271	11
Gago-Dominguez	2011	Bladder	USA	Caucasian	PB	TaqMan	High	217	189	43	449	278	210	43	531	0.706	12
Gago-Dominguez	2011	Bladder	China	Asian	PB	TaqMan	High	178	236	85	499	203	270	54	527	0.009	12
Ding	2011	HCC	China	Asian	HB	TaqMan	Low	500	563	210	1273	526	604	198	1328	0.255	9
Chen	2011	Glioma	China	Asian	HB	MassARRAY	High	351	461	141	953	430	486	117	1033	0.246	11
Liu	2011	SCCHN	USA	Caucasian	HB	TaqMan	Low	481	351	56	888	468	356	61	885	0.546	9
Xu	2012	Gastric	China	Asian	HB	PCR-RFLP	High	116	130	51	297	119	137	50	306	0.322	10
Hofer	2012	Colorectal	Austria	Caucasian	PB	TaqMan	High	86	45	6	137	963	623	119	1705	0.186	12
Wang	2012	Cervical	China	Asian	PB	TaqMan	High	375	444	174	993	397	480	138	1015	0.710	12
Li	2013	Lung	China	Asian	HB	TaqMan	High	173	207	88	468	227	250	67	544	0.886	10
Ma	2013	Bladder	China	Asian	PB	MassARRAY	High	71	75	28	174	373	461	127	961	0.408	12
Sheng	2013	ALL	China	Asian	PB	TaqMan	High	236	238	93	567	276	298	96	670	0.286	14
Wu	2013	Lung	China	Asian	HB	TaqMan	Low	205	232	102	539	263	278	86	627	0.361	8
Zhang	2013	HCC	China	Asian	HB	PCR-RFLP	Low	133	206	61	400	177	158	65	400	0.004	9
Gao	2014	Lung	China	Asian	HB	MassARRAY	Low	122	145	42	309	137	143	28	308	0.104	7
Hashemi	2014	Breast	Iran	Asian	PB	PCR-RFLP	Low	72	140	40	252	51	113	58	222	0.777	7
Singh	2014	Bladder	India	Asian	HB	TaqMan	High	77	106	42	225	117	95	28	240	0.203	9
Su	2014	HCC	China	Asian	HB	TaqMan	Low	75	97	29	201	111	76	23	210	0.077	8
Yin	2014	Esophageal	China	Asian	HB	PCR	High	245	277	78	600	270	306	75	651	0.403	11
Zhang	2014	Lung	China	Asian	HB	PCR	High	135	173	58	366	157	171	36	364	0.283	10
Zhao	2014	Lung	China	Asian	HB	TaqMan	High	337	438	177	952	406	443	106	955	0.365	12
Campa	2015	Pancreatic	Mixed	Caucasian	PB	TaqMan	Low	980	584	126	1690	1839	1307	251	3397	0.372	9
Jannuzzi	2015	Colorectal	Turkey	Caucasian	HB	PCR-RFLP	Low	25	14	65	104	15	28	92	135	0.000	9
Yoo	2015	Lung	Korea	Asian	HB	FIHP	Low	499	465	130	1094	487	472	98	1057	0.283	9
De Martino	2016	RCC	Austria	Caucasian	HB	ES	Low	24	123	92	239	121	151	94	366	0.001	5
Oztas	2016	Breast	Turkey	Caucasian	HB	PCR-RFLP	Low	40	52	15	107	26	62	20	108	0.115	9
Xing	2016	Lung	China	Asian	HB	TaqMan	Low	210	161	47	418	264	123	23	410	0.092	8
Lu	2016	Bladder	China	Asian	HB	PCR-RFLP	Low	58	95	48	201	80	88	32	200	0.349	8
Carkic	2016	OSCC	Serbia	Caucasian	HB	PCR-RFLP	Low	38	45	7	90	15	73	12	100	0.000	6
Xiao	2017	Lung	China	Asian	HB	TaqMan	Low	78	95	30	203	123	77	25	225	0.020	7
Yuan	2017	HCC	China	Asian	HB	TaqMan	Low	85	127	19	231	94	115	31	240	0.650	7

HWE, Hardy-Weinberg equilibrium; OSCC, oral squamous cell carcinoma; RCC, renal cell carcinoma; SCCHN, squamous cell carcinoma of head and neck; HCC, hepatocellular carcinoma; ALL, acute lymphoblastic leukemia; PB, population based; HB, hospital based; PCR, polymerase chain reaction; PCR-RFLP, polymerase chain reaction-restriction fragment length polymorphism; FIHP, Fluorescence-labeled hybridization probes; ES, electrophoretic separation.

### Meta-analysis results

**Figure 2:**
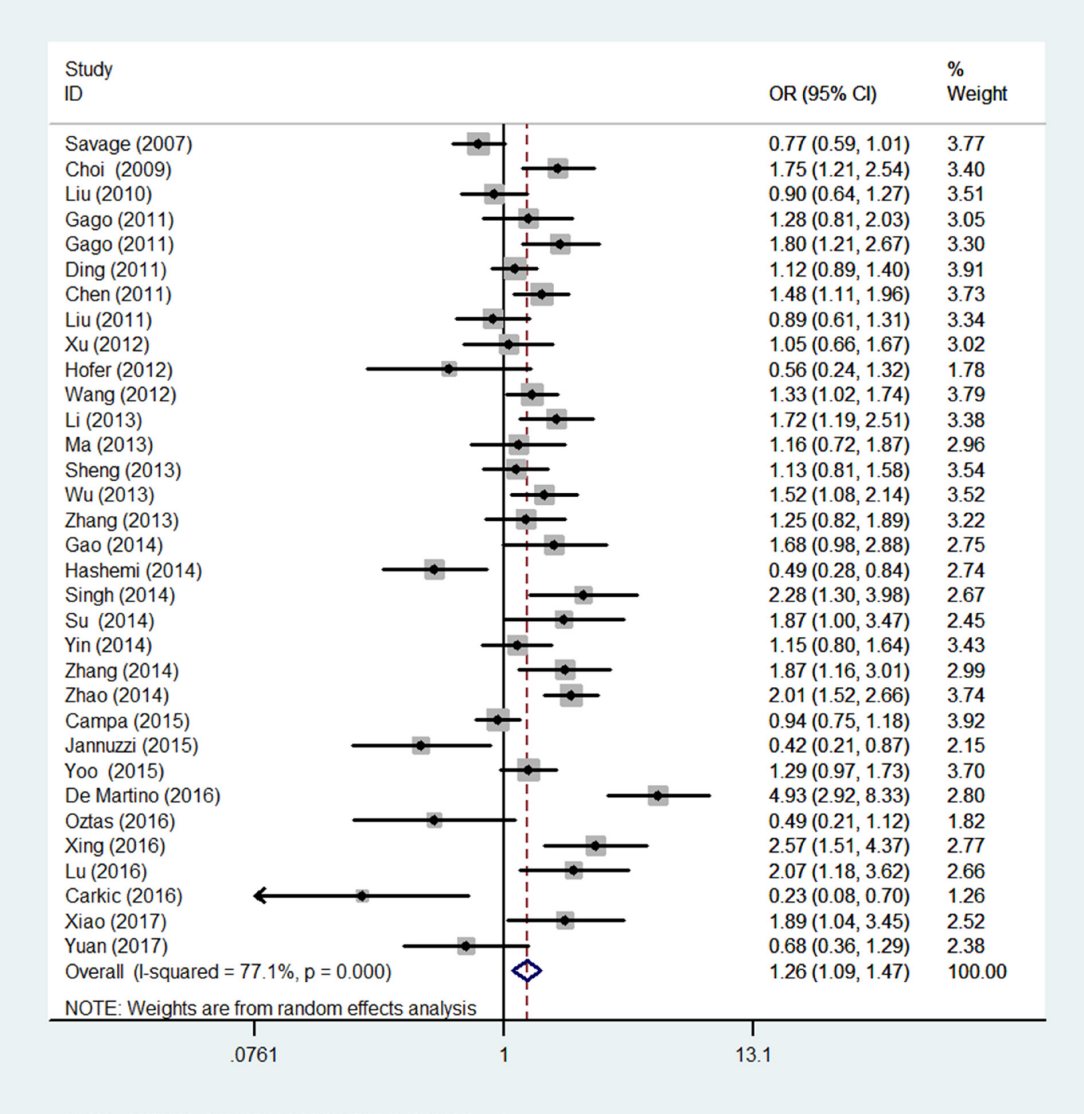
Forest plot of TERT rs2736098 polymorphism and overall cancer susceptibility (allele comparison model) The horizontal lines represent the study-specific ORs and 95% CIs, respectively. The diamond represents the pooled results of OR and 95% CI. The random effect model generates a constant from the homogeneity statistic Cochran's Q and using this and other study parameters a random effects variance component is generated. The inverse of the sampling variance plus this constant that represents the variability across the population effects is then used as the weight.

We presented the detailed results of association between rs2736098 polymorphism and cancer risk in Table 2 and Figure 2. Overall, we detected significant association between rs2736098 polymorphism and cancer risk among four genetic models (AA vs. GG: OR = 1.26, 95% CI = 1.09–1.47; AA vs. AG/GG: OR = 1.22, 95% CI = 1.09–1.36; AA/AG vs. GG: OR = 1.13, 95% CI = 1.02–1.24; A vs. G: OR = 1.11, 95% CI = 1.04–1.20). Stratification analysis by cancer type revealed that statistically significantly increased risk was found among lung cancer, bladder cancer, breast cancer, colorectal cancer, and other cancers, but not HCC and SCCHN. Further subgroup analysis by ethnicity, a significantly increased cancer risk was observed in Asians in all genetic models, but not Caucasians. As to the subgroup of control source, only hospital-based subgroup could contribute to increase risk of cancer. When stratified by quality score, significantly increased risk was observed in the score > 9 group, but not ≤ 9 group. We also observed significantly increased risk in subgroup of those SNP of controls agreement with HWE in all genetic models tested (AA vs. GG: OR = 1.25, 95% CI = 1.08–1.43; AA vs. AG/GG: OR = 1.21, 95% CI = 1.08–1.36; AA/AG vs. GG: OR = 1.10, 95% CI = 1.01–1.19; A vs. G: OR = 1.10, 95% CI = 1.03–1.18), with the exception of the heterozygote comparison.

**Table 2:** Meta-analysis of the association between *TERT* rs2736098 polymorphism and overall cancer risk

Variables	No. of	Homozygous			Heterozygous			Recessive			Dominant			Allele	
	studies	AA vs. GG			AG vs. GG			AA vs. AG/GG			AA/AG vs. GG			A vs. G	
		OR (95% CI)	*P* het		OR (95% CI)	*P* het		OR (95% CI)	*P* het		OR (95% CI)	*P* het		OR (95% CI)	*P* het
All ^a^	33	1.26 (1.09–1.47)	< 0.001		1.09 (0.99–1.19)	< 0.001		1.22 (1.09–1.36)	< 0.001		1.13 (1.02–1.24)	< 0.001		1.11 (1.04–1.20)	< 0.001
Cancer type															
Lung	9	1.71 (1.51–1.94)	0.453		1.18 (1.05–1.34)	0.037		1.60 (1.42–1.80)	0.783		1.29 (1.14–1.46)	0.021		1.29 (1.18–1.41)	0.033
Bladder	5	1.62 (1.27–2.08)	0.258		1.17 (0.93–1.45)	0.068		1.52 (1.24–1.85)	0.569		1.26 (1.01–1.57)	0.045		1.25 (1.08–1.45)	0.086
HCC	4	1.16 (0.87–1.54)	0.161		1.38 (0.97–1.95)	0.001		1.01 (0.78–1.30)	0.181		1.33 (0.98–1.79)	0.004		1.15 (0.97–1.36)	0.040
Breast	3	0.64 (0.46–0.89)	0.235		0.87 (0.68–1.13)	0.194		0.71 (0.57–0.88)	0.361		0.80 (0.59–1.07)	0.117		0.82 (0.67–0.99)	0.107
SCCHN	2	0.90 (0.70–1.16)	0.963		0.92 (0.81–1.05)	0.577		0.93 (0.73–1.20)	0.862		0.92 (0.81–1.04)	0.627		0.94 (0.85–1.03)	0.751
Colorectal	2	0.48 (0.28–0.83)	0.611		0.54 (0.21–1.40)	0.047		0.73 (0.46–1.14)	0.630		0.59 (0.31–1.12)	0.097		0.72 (0.56–0.92)	0.371
Others	8	1.26 (0.92–1.73)	< 0.001		1.02 (0.80–1.30)	< 0.001		1.23 (1.06–1.42)	0.121		1.06 (0.83–1.36)	< 0.001		1.08 (0.91–1.27)	< 0.001
Ethnicity															
Asians	23	1.43 (1.26–1.63)	< 0.001		1.15 (1.05–1.25)	< 0.001		1.33 (1.19–1.50)	0.001		1.21 (1.11–1.32)	< 0.001		1.19 (1.12–1.27)	< 0.001
Caucasians	10	0.88 (0.61–1.25)	< 0.001		0.90 (0.72–1.11)	< 0.001		0.97 (0.80–1.17)	0.024		0.90 (0.72–1.11)	< 0.001		0.93 (0.80–1.08)	< 0.001
Source of control															
HB	24	1.38 (1.16–1.65)	< 0.001		1.16 (1.02–1.31)	< 0.001		1.29 (1.15–1.50)	0.001		1.20 (1.06–1.37)	< 0.001		1.17 (1.07–1.28)	< 0.001
PB	9	1.03 (0.82–1.29)	< 0.001		0.93 (0.87–0.99)	0.549		1.05 (0.85–1.31)	< 0.001		0.96 (0.88–1.04)	0.164		0.99 (0.90–1.09)	0.002
Quality score															
> 9	15	1.30 (1.10–1.54)	< 0.001		1.02 (0.96–1.08)	0.530		1.29 (1.11–1.49)	0.001		1.07 (0.99–1.16)	0.021		1.10 (1.02–1.19)	< 0.001
≤ 9	18	1.23 (0.96–1.57)	< 0.001		1.15 (0.96–1.39)	< 0.001		1.15 (0.98–1.35)	< 0.001		1.17 (0.97–1.41)	< 0.001		1.12 (0.99–1.28)	< 0.001
HWE in controls															
Yes	27	1.25 (1.08–1.43)	< 0.001		1.05 (0.98–1.12)	< 0.001		1.21 (1.08–1.36)	< 0.001		1.10 (1.01–1.19)	< 0.001		1.10 (1.03–1.18)	< 0.001
No	6	1.23 (0.62–2.44)	< 0.001		1.08 (0.58–2.02)	< 0.001		1.22 (0.87–1.71)	0.009		1.13 (0.62–2.04)	< 0.001		1.12 (0.80–1.56)	< 0.001

HWE, Hardy–Weinberg equilibrium; Het, heterogeneity; HCC, hepatocellular carcinoma; SCCHN, squamous cell carcinoma of head and neck; HB, hospital based; PB, population based.

### Heterogeneity and sensitivity analysis

**Figure 3:**
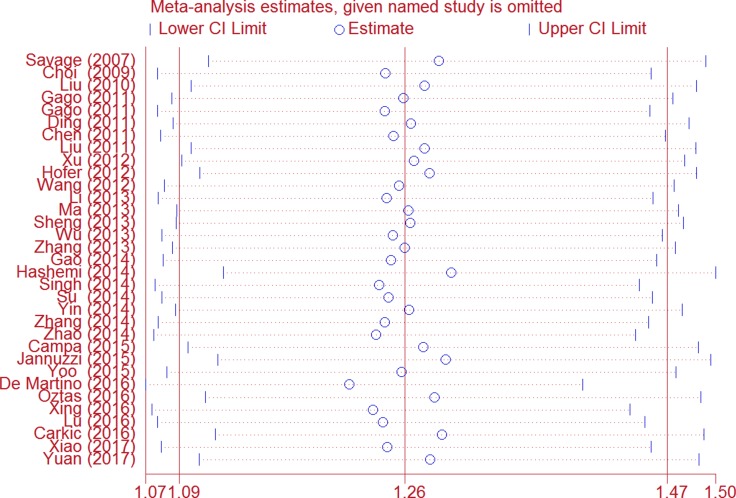
Sensitivity analysis of the association between *TERT* rs2736098 and cancer risk (allele comparison model) Each point represents the recalculated OR after omitting a separate study.

We first conducted *Q* test and *I*^2^ statistics to test between-study heterogeneity. Heterogeneity was indicated among all five genetic models as *P* < 0.001. Thus, the random-effect model was employed to generate wider CIs. As to the sensitivity analysis, the leaving each study out strategy showed that no substantial changes in ORs were observed after omitting each study (Figure 3). This reflects the stability and reliability of this meta-analysis.

### Publication bias

**Figure 4:**
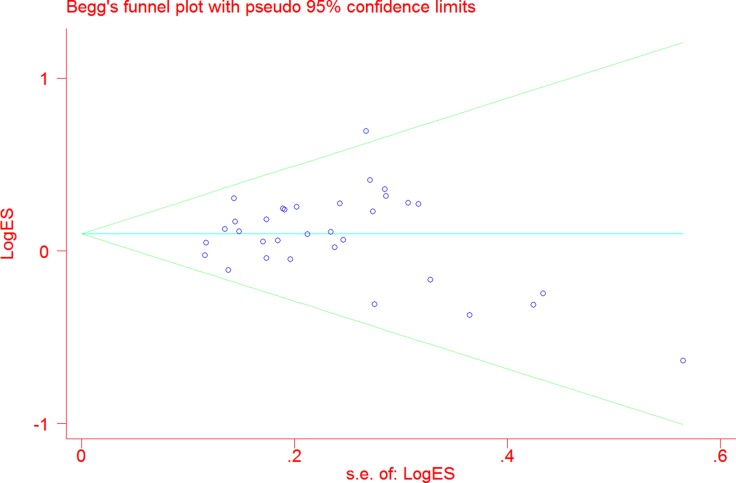
Funnel plot analysis to evaluate publication bias for TERT rs2736098 polymorphism (allele comparison model) Each point represents a separate study.

In Begg's funnel plots, we could not detect any obvious asymmetrical shape (Figure 4). Moreover, Egger's test result also revealed no evidence of publication bias among the studies (AA vs. GG: *P* = 0.92; AG vs. GG: *P* = 0.16; AA vs. AG + GG: *P* = 0.52; AA + AG vs. GG: *P* = 0.16; and A vs. G: *P* = 0.34).

## DISCUSSION

In this meta-analysis, we attempted to settle down the debate about the role of *TERT* rs2736098 in cancer risk. The obtained results suggested that there exists a significant relationship between *TERT* rs2736098 and cancer risk. To the best of our knowledge, this updated meta-analysis involves the largest samples and the most convincing conclusions.

Numerous studies have investigated the role of *TERT* gene rs2746098 polymorphism in the contributions to cancer risk. To obtain a clear association between *TERT* rs2736098 and cancer risk, several meta-analyses have been performed. The first meta-analysis was conducted by Zhang et al. [14] in 2012, with 8 studies consisting of 8,070 cases and 10,239 controls. They claimed that no significant association was observed between *TERT* rs2736098 polymorphism and overall cancer risk. However, after stratified by ethnicity, a significantly increased risk of cancers was shown Among Asians. In another meta-analysis with 12 studies including 10044 cases and 12480 controls. Wu et al. [16] found that there was a borderline significant increased overall cancer risk conferred by rs2736098. In addition, such increased cancer risk was more obvious among lung cancer, bladder cancer, hospital-base design and Asians. The most recent published meta-analysis included 19 studies with 12520 cases and 14968 controls [18]. They found that GA/AA variant could contribute to increased risk of overall cancer. Their stratification analysis revealed that such association was more significant in Asians, lung cancer and hepatocellular carcinoma. It is obvious that conflict conclusions still exist, due to the relative small sample size included.

As several new studies have been updated since the latest meta-analysis, it is necessary for us to incorporate all the accessible studies to better elucidate the association between *TERT* rs2736098 polymorphism and cancer risk. In all, we found a significant relationship between *TERT* rs2736098 and cancer risk in the pooled analysis under all the five-genetic model, except for heterozygous model. Such findings were consistent with the results reported in the study of Wu et al. [16] and the latest meta-analysis [18]. Subgroup analysis by ethnicity suggested that individuals carrying *TERT* rs2736098 polymorphism from Asians but not among Europeans were more likely to exhibit an increased cancer risk, possibly because of the differences in genetic backgrounds among different populations. Our results suggested that genetic variants in *TERT* significantly increased the risk of lung cancer, bladder cancer, breast cancer, colorectal cancer and other cancer, but not HCC and SCCHN. These lines of evidence suggested that *TERT* rs2736098 polymorphism may have different effects in different cancer types. The possible reasons for discrepancies regarding cancer susceptibility may be ascribed to tumor specificity, differences in ethnicity, and variations in sample sizes included in each investigation.

To improve the quality of the current meta-analysis, we adopted some measurements below. First, our meta-analysis was the first to search literatures from both English and Chinese, with the aim to strengthen the reliability of our conclusions. Second, we adopted sensitivity analysis and publication bias assay, and the results indicated that the conclusions are robust and no publication bias was detected.

Yet, some limitations still exist and thus cautions are needed before interpreting the results obtained from the current meta-analysis. First, the number of included studies is far from enough to obtain a robust conclusion, especially for stratified analysis. Second, the validity of conclusion might be discount as significant between-study heterogeneity was observed in some comparisons. Third, we only calculated the crude ORs, but not the adjusted ORs, due to the lack of other important information like environmental factors, age, drinking status, and gene-environment interactions. Fourth, selection bias could not be avoided, as only the studies written in English or Chinese were extracted. Last, nearly all the eligible case-control studies included were conducted among Asians and Caucasians, other ethnicities such as Africans were not undertaken. Concerning genetic and geographical differences, additional studies are needed to further confirm such conclusion from other ethnicities, especially Africans.

To sum up, the current meta-analysis provides a powerful evidence that *TERT* rs2736098 polymorphism is associated with cancer risk, from the perspective of the formed case-control studies. However, it is still needed for us to continue providing more new evidence based on large sample size, multi-center investigation case-control studies.

## MATERIALS AND METHODS

### Publication search

A comprehensive literature search was first conducted in English electronic database PubMed and EMBASE using the combination of the following items: “polymorphism or single nucleotide polymorphism or SNP or variant” and “*TERT* or *hTERT* or rs2736098 or telomere reverse transcriptase”, and “cancer or neoplasm or tumor or carcinoma”. Then we further expanded the searching field to Chinese database China National Knowledge Infrastructure (CNKI) and Wanfang database using the same combination items in Chinese. The searching time was updated to August 2017. Moreover, we also included the eligible studies extracted from the references of retrieved articles. A single study would be treated as separate studies if two more ethnic subpopulations is included. Only the largest or the latest study was included if there exist two more articles with overlapping data. No language publication restrictions were set in this searching strategy. The designation and writing of this meta-analysis was under the guidelines of Preferred Reporting Items for Systematic Reviews and Meta-analyses.

### Eligibility criteria

In the current meta-analysis, only the studies met the following criteria were included: (1) studies published in English or Chinese; (2) unrelated case-control studies; (3) tested for the association of TERT rs2736098 polymorphism with cancer risk; (4) enough information to calculate odds ratios (ORs) and 95% confidence intervals (CIs). Studies that failed to meet the above criteria were excluded in the final analysis.

### Data extraction

We arranged two authors (Tingyuan and Minjie) to screen the articles and extracted available data from all eligible studies, blindly. The data shown below were extracted: first author's surname, publication year, country, ethnicity, the source of controls, genotyping methods, quality score, and numbers of cases and controls with AA, AG and GG genotypes. Any discrepancy was resolved after full discussion.

### Quality assessment

To strengthen the robustness of our meta-analysis, a quality assessment was performed to all the included studies through adopting the quality assessment criteria (Supplementary Table 1). In brief, the quality scores range from 0 to 15. The studies with a score less than 9 were classified as low quality, while those more than 9 were classified as high quality.

### Statistical methods

We first adopted goodness-of-fit χ^2^ test to assess whether the SNP in the control was departure from HWE. The strength of the association between *TERT* rs2736098 polymorphism and overall cancers risk was measured by calculating crude ORs and their 95% CIs using all five genetic models: homozygous model (AA vs. GG), heterozygous model (AG vs. GG), recessive model (AA vs. AG + GG), dominant model (AA + AG vs. GG) and allele comparison (A vs. G). Stratification analyses were also performed by ethnicity, and source of control, quality score, and HWE in controls. Between-study heterogeneity was analyzed by the Cochran's *Q* test and quantified by *I*^2^ statistics. When homogeneity existed, the fixed model (Mantel-Haenszel method) was used to calculate the summary ORs and 95% CIs; otherwise, the random-effects model (the DerSimonian and Laird method) was utilized. Sensitivity analysis was done by individually removing studies one by one and reanalyzing the pooled risk estimates. Publication bias was further assessed using Begg's funnel plot and Egger's linear regression, with that asymmetric plot and a *P value* < 0.05 indicating the presence of publication bias. All statistical analysis was completed using STATA software (Stata Corporation, College Station, TX; version 11.0). All the statistics were two-sided with significant findings set at a *P value* of < 0.05.

## SUPPLEMENTARY MATERIALS FIGURES AND TABLES


