# Supplementary material for: TERT rs2736098 (Ex2-659G>A) polymorphism and cancer susceptibility: evidence from a comprehensive meta-analysis
Source: Oncotarget. 2017 Oct 9;8(56):96433–41. doi: 10.18632/oncotarget.21703 (PMC5707112; doi:10.18632/oncotarget.21703)
Supplement: Supplementary file 1 [file oncotarget-08-96433-s001.pdf]

TERT rs2736098 (Ex2-659G>A) polymorphism and cancer susceptibility: evidence from a comprehensive meta-analysis

SUPPLEMENTARY MATERIALS

Supplementary Table 1: Score of quality assessment

Criteria	Score
Representativeness of case	
Selected from population cancer registry	2
Selected from hospital	1
No method of selection described	0
Representativeness of control	
Population-based	3
Blood donors	2
Hospital-based	1
Not described	0
Ascertainment of cancer cases	
Histopathologic confirmation	2
Patient medical record	1
Not described	0
Control selection	
Controls matched with cases by age and sex	2
Controls matched with cases only by age or by sex	1
Not matched or not descried	0
Genotyping examination	
Genotyping done blindly and quality control	2
Only genotyping done blindly or quality control	1
Unblinded and without quality control	0
HWE	
HWE in the control group	1
HWE in the control group or not mentioned	0
Total sample size	
> 1000	3
501–1000	2
201–500	1
≤ 200	0
